# Isolated Lupus-Associated Protein-Losing Enteropathy in a Resource-Limited Centre

**DOI:** 10.7759/cureus.15826

**Published:** 2021-06-22

**Authors:** Kasun Prabasara, K T Sundaresan, Chamith Rosa

**Affiliations:** 1 Medicine, Teaching Hospital, Batticaloa, LKA; 2 Clinical Sciences, Eastern University of Sri Lanka, Batticaloa, LKA

**Keywords:** isolated lupus associated protein losing enteropathy, luple, sle, generalized body swelling, hypoalbuminemia

## Abstract

Systemic lupus erythematosus (SLE) is a relatively common autoimmune disease with recently reported cases of lupus-associated protein-losing enteropathy (LUPLE) as an unusual manifestation. It is a well-recognized clinical entity predominantly affecting middle-aged Asian females. LUPLE is diagnosed by exclusion of possible causes for hypoalbuminemia in a patient with positive anti-nuclear antibody (ANA). LUPLE as the first manifestation of SLE is rare but it is a well-recognized complication secondary to SLE. We report a case of a 39-year-old Sri Lankan lady who was investigated for generalized body swelling, pleural effusions, ascites and pericardial effusions due to hypoalbuminemia. Her ANA was positive with speckled pattern and intestinal biopsy samples revealed evidences of chronic inflammatory cell infiltrates in laminapropria. Her investigations were not suggestive of liver diseases, albuminuria or malnutrition. We excluded all possible etiologies for protein-losing enteropathy although gold standard tests to confirm it was not available in our center. In conclusion, LUPLE should be considered as an etiology for all the unexplained protein-losing enteropathies. We suggest to treat LUPLE with prednisolone, hydroxychloroquine (HCQ) followed by steroid-sparing agents such as azathioprine. Prognosis was excellent following appropriate treatment.

## Introduction

Systemic lupus erythematosus (SLE) is a multisystem disorder secondary to autoimmune aetiology which causes widespread inflammation and tissue damage in affected organs [1]. Gastrointestinal manifestations were reported in up to 40% of patients with diagnosed SLE [2]. These manifestations are described secondary to vasculitides, lymphangiectasia, adverse drug effects and secondary to infections. Esophageal dysmotility, peptic ulcers, intestinal pseudo-obstructions and lupus-associated protein-losing enteropathy (LUPLE) were reported gastrointestinal manifestations of SLE [2]. Although LUPLE was recognized as a complication of SLE, there were latest case reports that state it as the isolated initial manifestation of SLE [3,4]. Severe diarrhea was reported in 50% of patients with LUPLE. But generalized body swelling, ascites and effusions were usual manifestations of the remainder [2]. Liver diseases causing reduced albumin production, nephrotic range proteinuria and malnutrition due to various causes has to be excluded before concluding diagnosis as protein-losing enteropathy [2]. LUPLE is a diagnosis of exclusion of all other possible causes for protein-losing enteropathies [2]. snti-nuclear antibody (ANA) was positive in almost all patients (96%), predominantly speckled pattern was observed [5]. Technetium-99m albumin scintigraphy and Alpha-1-Antitrypsin clearance were technically used to confirm protein loss through the gastrointestinal tract [2]. A systemic review was performed with 112 LUPLE patients and outcomes were as below [5]. Intestinal imagings were normal in 52% of cases and 44% showed colonic thickening. Hypocomplementemia was seen in 79% of total cases. Intestinal biopsies revealed inflammatory cell infiltrates, mucosal thickening, vasculitides or lymphangiectasia in 80% of patients. All the patients that were included in the systemic review had started on steroids. 34% of them were responded to prednisolone alone, 68% needed combined immunosuppressant but the overall prognosis was excellent after appropriate treatments [5]. We present here a case of a 39-year-old Sri Lankan lady who had presented to us with generalized body swelling, diagnosed as LUPLE in a low resource center.

## Case presentation

A 39-year-old Sri Lankan lady from a rural area presented to our tertiary care centre with generalized body swelling for one-week duration. Her history was started one year and three months before with the swelling of both ankles which was more prominent towards the latter part of the day. Swelling of the periorbital area and both hands in subsequent days prompted her to seek medical attention. Despite multiple therapeutic efforts by general practitioners (GPs), her ankle swelling kept worsening and this was followed by abdominal swelling. Then she was referred to our tertiary care institution by a GP for further evaluation of her body swelling. She also had noticed to have increased hair loss for the last 3-months. There was no history of Raynaud’s phenomenon, photosensitive rash, oral ulcers, dry eyes, and dry mouth or pregnancy losses. She had no history of nausea, vomiting, body itching or frothy urine. There was no history of jaundice, malaena or long-term use of alcohol-containing drinks. She had no orthpnoea, paroxysmal nocturnal dyspnoea (PND) or chest pain. She had no symptoms suggestive of hypothyroidism. There was no history of diarrhea, blood-stained stools or recurrent abdominal pain. She used to take a traditional nonvegetarian Sri Lankan diet with adequate nutrition supplementations. She was completely normal in her past without any significant medical conditions. She had no significant family history or known allergies.

On examination, she was oedematous and her body weight was 65 kg. The swelling was predominantly in the ankles and it was pitting in nature. She was afebrile, not pale or icteric. There were no skin rashes, oral ulcers, alopecia, thickened skin or vasculitic rashes. There was no clubbing or features of arthritis. There were no peripheral stigmata to suggest chronic liver disease (CLD) or chronic kidney disease (CKD). Her respiratory examination consisted of bilateral pleural effusions. Pulse rate was 102/min and blood pressure was 100/62 mmHg. Heart sounds appeared muffled on auscultation. Abdomen was distended and there was clinical evidence of gross ascites without organomegaly. Nervous system examination was normal.

Her full blood count (FBC), serum creatinine (S.Cr.), blood urea (BU), serum electrolyte (SE), serum calcium (Ca), serum magnesium (Mg) and urine full report (UFR) were entirely normal. Other investigations were as follow (Table 1).

**Table 1:** Investigations. SGOT: serum glutamic oxaloacetic transaminase; SGPT: serum glutamic-pyruvic transaminase; ALP: alkaline phosphatase; ANA: anti-nuclear antibody; IgM: immunoglobulin M; IgA: immunoglobulin A; IgG: immunoglobulin G; CEA: carcinoembryonic antigen; CRP: C-reactive protein; ESR: erythrocyte sedimentation rate; RBC: red blood cell; TSH: thyroid-stimulating hormone.

Investigation	Value	Investigation	Value
SGOT (8-45 U/L)	46 U/L	T4 (4.6-12 ug/dl)	6 micro-gram/dl
SGPT (7-56 U/L)	48 U/L	TSH (0.4-4 mIU/L)	2.1 mIU/L
ALP (44-140 U/L)	58 U/L	Urine albumin creatinine ratio (ACR) (<1 mg/mmol)	0.5 mg/mmol
Gamma-GT (0-30 IU/L)	32 IU/L	9.00 am Cortisol (> 420 nmol/L)	510 nmol/L
Total bilirubin (5.1-17 umol/L)	8.2 umol/L	ANA (<1: 80)	>1:100 positive
Total protein (6-8.3 mg/dl)	5.1 mg/dl	C3 (55-120 mg/dl)	82 mg/dl
Albumin (3.4-5.4 mg/dl)	1.2 mg/dl	C4 (20-50 mg/dl)	29 mg/dl
Globulin (2-2.9 mg/dl)	3.9 mg/dl	B12 level (190-950 pg/ml)	202 pg/ml
ESR	76 mm/1^st^ hour	RBC folate level (140-628 ng/ml)	161 ng/ml
CRP (< 6 mg/dl)	5 mg/dl	Stool calprotectin	Negative
CA 125 (<46 U/ml)	484 U/ml	Endomysial antibody	Negative
CEA (0-2.5 ng/ml)	1 ng/ml	Tissue transglutaminase antibody	Negative
IgM (41-147 mg/dl)	155 mg/dl	IgA (61-330 mg/dl)	123 mg/dl
IgG (566-1919 mg/dl)	556 mg/dl		

Pleural fluid and peritoneal fluid were sent for full report (cell count and differentials, protein, sugar), acid-fast bacilli (AFB) staining, gram stain and bacterial cultures. It was a transudative effusion and all the other screenings were normal.

**Figure 1:**
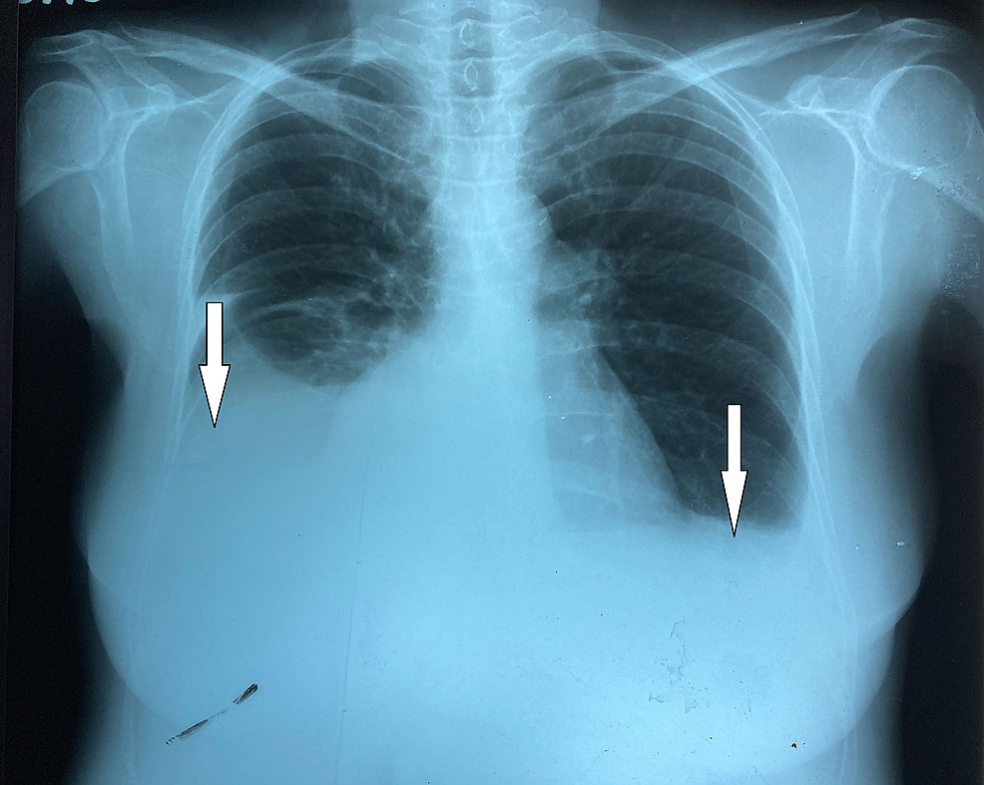
Chest X-ray PA shows bilateral pleural effusions. PA: posteroanterior.

Chest X-ray (Figure 1) revealed bilateral pleural effusions.

**Figure 2:**
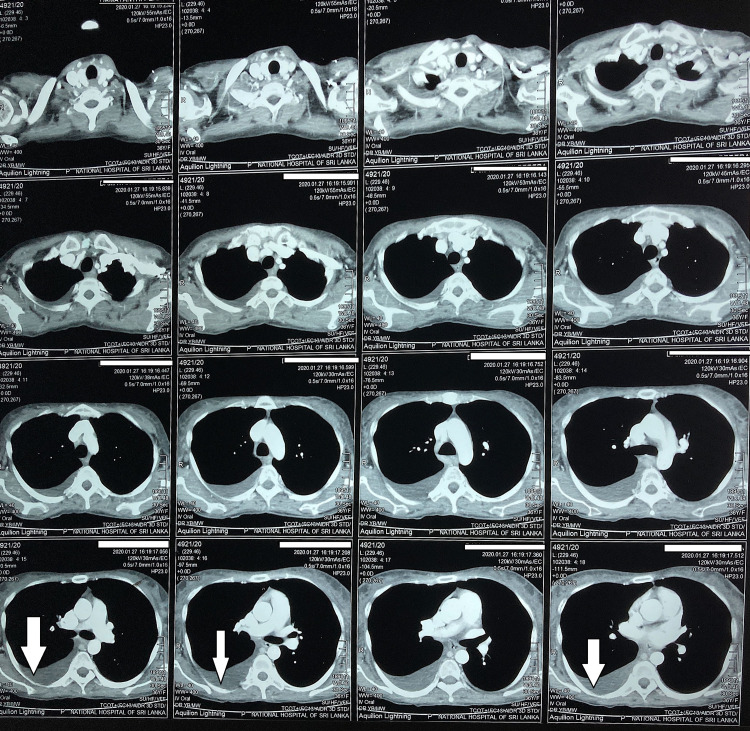
CT chest, abdomen and pelvis show right-sided pleural effusion.

**Figure 3:**
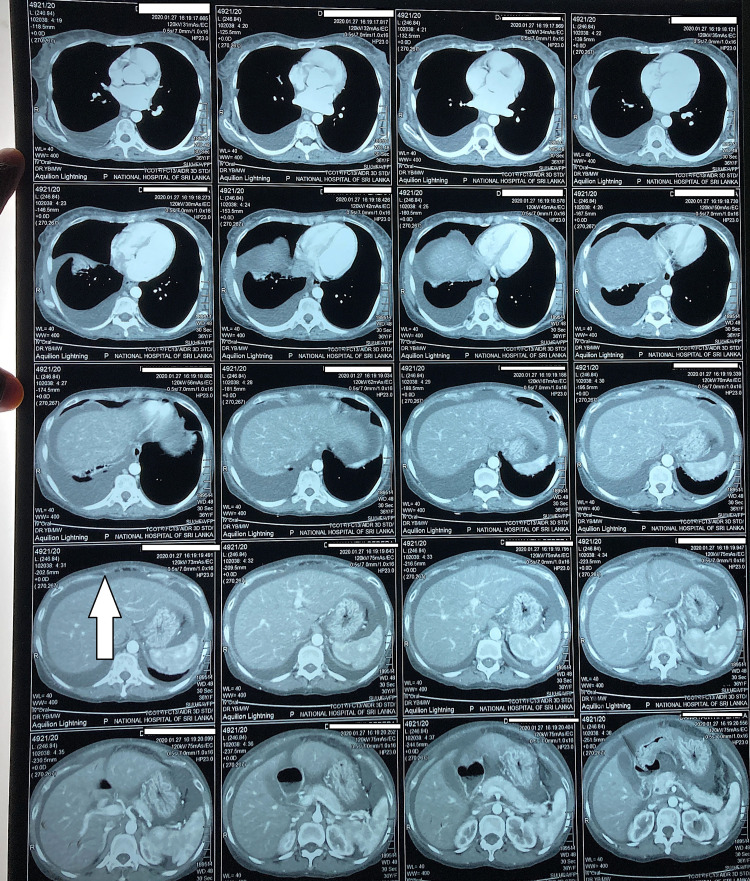
CT chest, abdomen and pelvis. Ascites fluid infront of liver.

**Figure 4:**
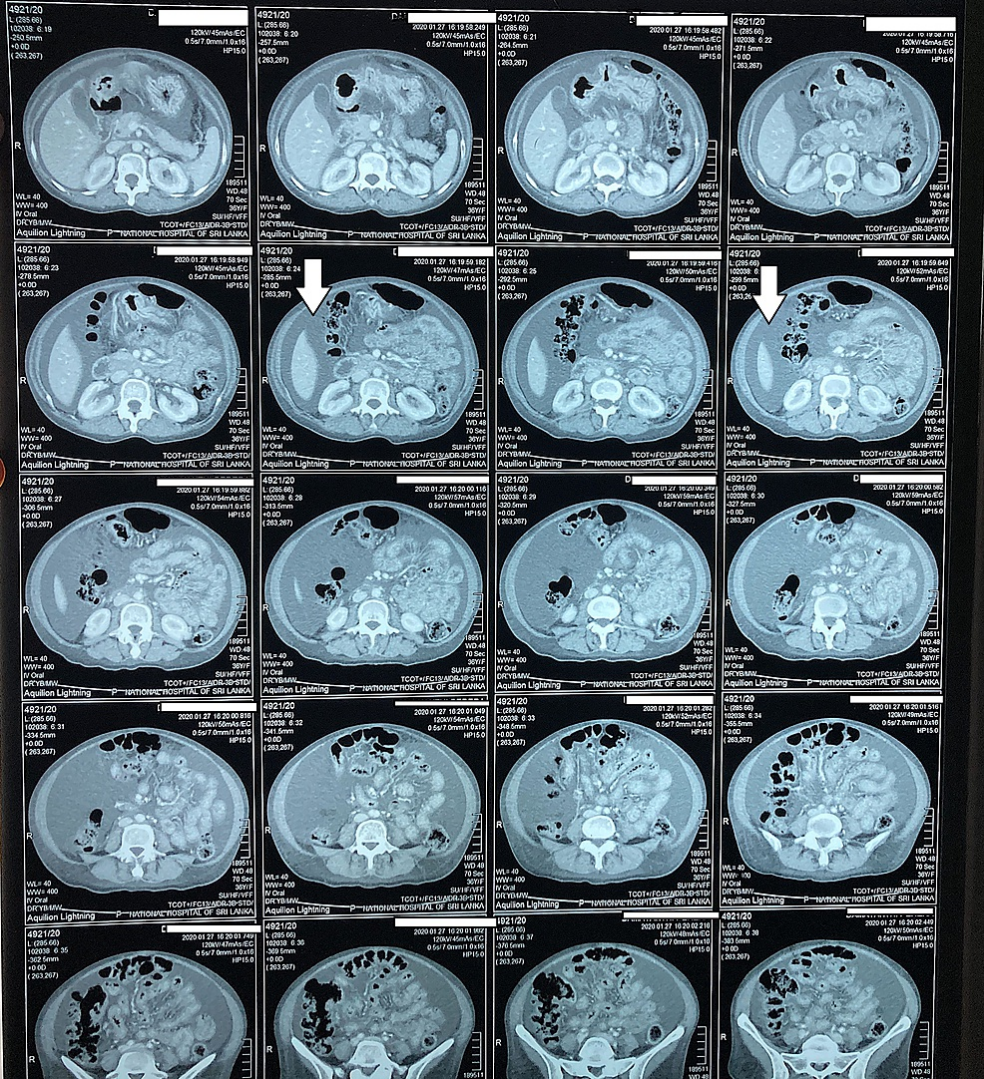
CT chest, abdomen and pelvis shows gross ascites.

CT of chest, abdomen and pelvis (Figures 2, 3, 4) revealed bilateral pleural effusions, ascites and mild pericardial effusion. 

2D echo showed preserved ejection fraction, normal valves and mild to moderate amount of pericardial effusion. Indirect immunofluorescence test (IIF) of anti-nuclear antibody became positive with a titre of >1:100 and fine speckled pattern was identified but anti-double-stranded DNA was negative (other autoantibodies screening did later and has mentioned in table 2). Serum protein electrophoresis did not reveal abnormal monoclonal bands. Duodenal biopsy was specially looked for possible evidences of celiac disease but those features were not seen and it revealed mild lymphoplasmacytic infiltrates. Jejunal biopsy showed aggregates of lymphocytes, forming lymphoid follicle in lamina propria with preserved intestinal architecture. Ileal biopsy and transverse colonic biopsies were normal.

**Figure 5:**
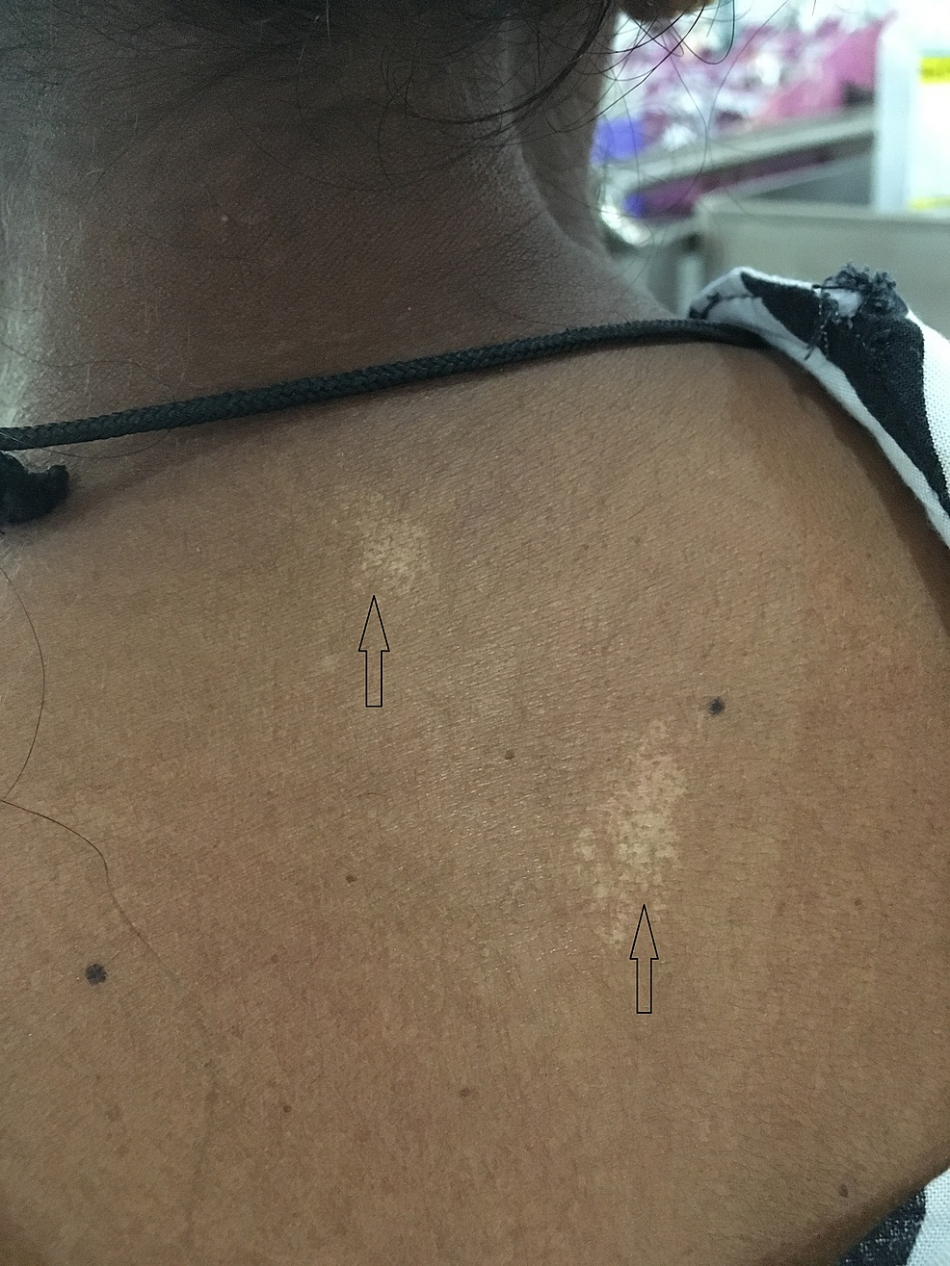
Two patches of speckled leukoderma.

Following these extensive investigations, we managed her as idiopathic systemic capillary leaking syndrome (ISCLS) with five days of IV immunoglobulin followed by monthly IV immunoglobulin prophylaxis and discharged. Three weeks later she again admitted with same complain and very low albumin. We had to abandon ISCLS, which was our previous diagnosis, due to poor clinical and biochemical response following adequate prophylaxis with immunoglobulin treatment. It has been a year since she got admitted to our institution with the same complaint. Later few skin lesions were found behind the neck and those lesions were confirmed as patches of speckled leukoderma (Figure 5).

We repeated ANA immunofluorescence and it came as high titre positive (>1: 1280, fine speckled pattern). We arranged extractable nuclear antibodies (ENA) panel as autoantibody screening and all were negative (Table 2).

**Table 2:** Autoantibody screening.

Investigation	Result	Investigation	Result
Anti-U(1) RNP antibody	Negative	Anti-scl-70 Antibody	Negative
Anti Sm antibody	Negative	Anti-Jo-1 Antibody	Negative
Anti Ro Antibody	Negative	Beta 2 glycoprotein Antibody	Negative
Anti La antibody	Negative	Anti-phospholipid antibody	Negative

We could not arrange faecal Alpha-1-Antitrypsin clearance or Technetium-99m albumin scintigraphy due to unavailability of the above tests in our centre. Capsular endoscopy was arranged to visualize small intestine and it was completely normal.

We conducted three times multi-disciplinary team (MDT) discussions and after excluding all other possible causes for her presentation, we decided to manage as LUPLE. High-dose prednisolone (1 mg/kg) daily and hydroxychloroquine (HCQ) 100 mg twice a day dose were started and albumin was corrected up to 3.1 mg/dl before discharge. Same drug doses were continued for one month and she was reviewed after one month with repeat albumin level. On her review visit, she had clinically improved with serum albumin level of 3.0 mg/dl. Therefore we started to slowly taper down prednisolone dose after adding azathioprine as steroid-sparing agent. She was monitored for six months with monthly liver function tests including albumin and FBC. We could tail off prednisolone to 5 mg daily dose over this six-month period without further significant loss of albumin. Six months later her albumin was improved to 3.3 mg/dl with remission of her disease.

## Discussion

LUPLE is a diagnosis of exclusion of other possible causes of protein-losing enteropathies. It is important because it is an unusual manifestation of SLE and it can be easily missed by clinicians. A systemic review including 112 LUPLE patients has discovered all the patients were 20-48 years, female: male ratio 5.8:1 and Asian predominance pattern [5]. Our patient is being a 39-year-old Asian female, fits into these categories. We have our Sri Lankan case reports that were published in the last decade on various presentations of LUPLE [6, 7]. One is a 35-year-old female presented with diarrhea and progressive abdominal distention and the other patient is a 56-year-old male presented with clinical features suggestive of SLE together with hypoalbuminemia. Both patients were diagnosed as LUPLE as an uncommon manifestation of SLE [6,7]. In both cases, there were evidence of SLE. The first case with arthritis and photosensitive rash, the second case with discoid rash. But in our patient’s first presentation was generalized body swelling without other features to suggest SLE. Former systemic review also revealed generalized body swelling, pleural effusions, ascites and mild pericardial effusion in 21%-48% of patients [5]. Her full liver function tests including SGOT, SGPT, ALP, GGT, bilirubin and prothrombin time/international normalized ratio (PT/INR) all were normal except significant hypoalbuminemia. Since liver was normal radiologically with normal liver function tests we could safely exclude liver diseases in etiological screening. We did urine albumin creatinine ratio (ACR) twice and no albuminuria was detected. Satisfactory 24-hour dietary recall and normal calcium, phosphate, alkaline phosphate, PT/INR, B12 and RBC folate levels were against nutritional deficiencies. With the above findings, protein-losing enteropathy was the only possible explanation for persistent severe hypoalbuminemia. Although we could not confirm it due to the unavailability of Technetium-99m albumin scintigraphy and Alpha-1-Antitrypsin clearance tests in our center.

We did contrast-enhanced computerized tomography (CECT) of chest, abdomen and pelvis, upper gastrointestinal endoscopy (UGIE), colonoscopy and capsular endoscope to have a macroscopic view and for biopsy purposes. All were normal excluding the possibilities of erosive gastrointestinal diseases such as Crohn’s, ulcerative colitis (UC) and gastrointestinal malignancies. Coeliac serology and duodenal biopsy findings were not suggestive of coeliac disease. Since there were no syndromic features and additional areas with defective lymphatic circulation, primary lymphangiectasia was unlikely. We extensively looked into possible causes of secondary lymphangiectasia such as cardiomyopathy, tricuspid regurgitation, retroperitoneal lymphadenopathy, portal hypertension and lymphomas and exclude all. Former systemic review illustrated positive ANA in 96% of cases and 56% showed speckled pattern like in our patient. Same systemic review showed mucosal oedema, inflammatory infiltrates, lymphangiectasia or vasculitides in 80% of patients with LUPLE [5]. Our patient’s duodenal biopsy showed mild lymphoplasmacytic infiltration with preserved epithelial architecture. Her jejunal biopsy revealed preserved small intestinal architecture; lamina propria shows aggregates of lymphocytes, forming lymphoid follicles. These biopsy findings were suggestive of chronic inflammatory cells infiltrate which can be the possible pathology behind protein-losing enteropathy in our patient. Technetium-99m albumin scintigraphy can locate the area of albumin loss that can correlate with biopsy findings. As per 112 patients that involved in the systemic review we also started treatment with prednisolone and HCQ after baseline fundoscopy. Since the patient was significantly improved with steroids we could tail off it while adding azathioprine as a steroid-sparing agent.

We excluded all the possible causes for protein-losing enteropathies with extensive investigations. Although our patient's clinical criteria were not fitting to SLE, we did not have any other explanations for her protein-losing enteropathy. She is being a young, female of Asian origin with speckled leukoderma patches to suggest autoimmune rheumatic disease, with the presence of ANA high titre we decided to manage her as LUPLE as the first manifestation of SLE. Her dramatic response to prednisolone was supportive for our diagnosis. Although LUPLE needs aggressive immunosuppressive treatments, considering the chronicity of her illness we treated our patient with oral steroids. 

## Conclusions

LUPLE is an uncommon manifestation of a common disease predominantly reported among young Asian females with SLE. Isolated LUPLE can be present without usual SLE features. LUPLE is a diagnosis of exclusion of possible causes for hypoalbuminemia in a patient with positive ANA. Chronic inflammatory cell infiltrates may be a contributory pathological finding although the exact mechanism unknown. All the patients with LUPLE should be started on prednisolone and the prognosis following appropriate treatment is excellent.
